# Sphingosine 1-Phosphate Receptors: Do They Have a Therapeutic Potential in Cardiac Fibrosis?

**DOI:** 10.3389/fphar.2017.00296

**Published:** 2017-06-02

**Authors:** Ambra Vestri, Federica Pierucci, Alessia Frati, Lucia Monaco, Elisabetta Meacci

**Affiliations:** ^1^Department of Experimental and Clinical Biomedical Sciences “Mario Serio", Molecular and Applied Biology Research Unit, University of FlorenceFlorence, Italy; ^2^Interuniversity Institutes of MyologyFirenze, Italy; ^3^Department of Physiology and Pharmacology “Vittorio Erspamer", Sapienza University of RomeRome, Italy

**Keywords:** sphingosine 1-phosphate, sphingolipids, matrix metalloproteinases, cardiomyocytes, collagen accumulation, G-coupled receptor, cardiac fibrosis

## Abstract

Sphingosine 1-phosphate (S1P) is a bioactive lipid that is characterized by a peculiar mechanism of action. In fact, S1P, which is produced inside the cell, can act as an intracellular mediator, whereas after its export outside the cell, it can act as ligand of specific G-protein coupled receptors, which were initially named endothelial differentiation gene (Edg) and eventually renamed sphingosine 1-phosphate receptors (S1PRs). Among the five S1PR subtypes, S1PR1, S1PR2 and S1PR3 isoforms show broad tissue gene expression, while S1PR4 is primarily expressed in immune system cells, and S1PR5 is expressed in the central nervous system. There is accumulating evidence for the important role of S1P as a mediator of many processes, such as angiogenesis, carcinogenesis and immunity, and, ultimately, fibrosis. After a tissue injury, the imbalance between the production of extracellular matrix (ECM) and its degradation, which occurs due to chronic inflammatory conditions, leads to an accumulation of ECM and, consequential, organ dysfunction. In these pathological conditions, many factors have been described to act as pro- and anti-fibrotic agents, including S1P. This bioactive lipid exhibits both pro- and anti-fibrotic effects, depending on its site of action. In this review, after a brief description of sphingolipid metabolism and signaling, we emphasize the involvement of the S1P/S1PR axis and the downstream signaling pathways in the development of fibrosis. The current knowledge of the therapeutic potential of S1PR subtype modulators in the treatment of the cardiac functions and fibrinogenesis are also examined.

## Intracellular and Extracellular Actions of S1P

**FIGURE 1:**
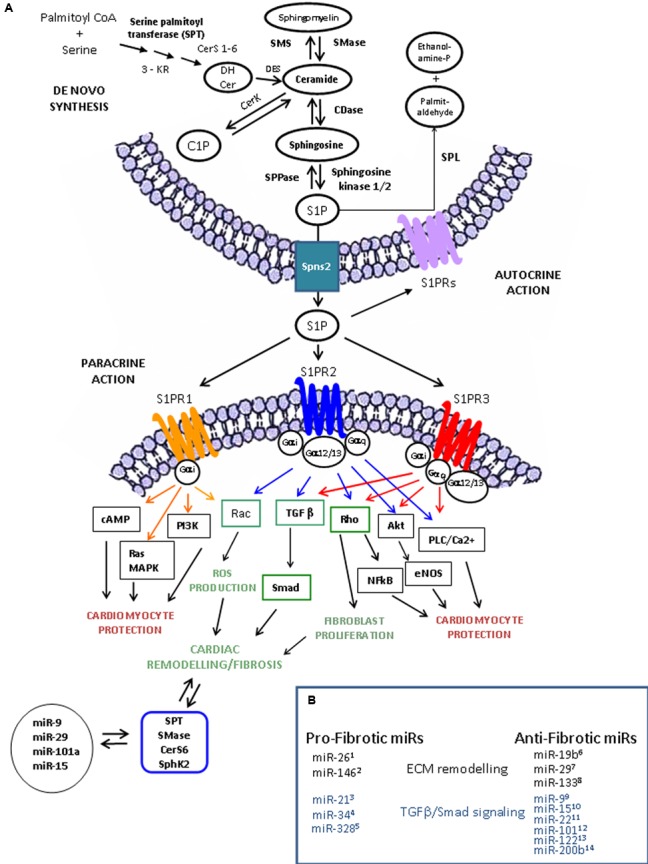
Sphingolipid metabolism, S1P receptor-activated pathways and miR involved in cardiac remodeling and functions. **(A)** S1P is synthesized from sphingosine (Sph) by the sphingosine kinases, SphK1 and SphK2 and irreversible cleaved by S1P lyase (SPL), which generates hexadecenal and phosphoethanolamine. S1P is also a substrate of specific S1P phosphatases (SPPase). Ceramide derives via the sphingomyelin cycle or *de novo* sphingolipid synthesis involving serine palmitoyl transferase (SPT), 3-keto reductase (3KR), ceramide synthase (CerS), and desaturase (DeS), and converted reversibly to sphingosine (Sph) by ceramidase (CDase), or phosphorylated to ceramide-1-phosphate (C1P) by ceramide kinase (CerK) activity. S1P produced inside the cell can be transported in the intercellular space by an ATP-binding cassette transporter named spinster homolog 2 (Spns2). As ligand, S1P acts as autocrine and paracrine factor triggering specific signaling pathways by interacting with S1P specific heterotrimeric GTP binding protein-coupled receptors (GPCR), named S1PR. Three among five subtypes of S1PRs, S1PR-1 (orange), -2 (blue), and -3 (red), are expressed in cardiomocytes, cardiac fibroblasts and procursor cardiac cells. In heart, S1PR activation leads to different cardiac effects (profibrotic, green; antifibrotic and cardioprotective, black/red). The scheme exemplifies, in accordance with the current literature, the main pathways triggered by S1PR activation leading to cardiac cell protection and extracellular matrix (ECM) remodeling. Interestingly, the expression of the key enzymes involved in sphingolipid metabolism can be regulated by microRNAs (miRs) and some of them (i.e., miR-9^9^, miR-19b^6^, miR-15^10^, and miR-29^7^) also regulate the fibrotic process by affecting extracellular matrix (ECM) remodeling through the modulation of metalloprotease (MMPs), TGF-β/TGFR and Smad protein expression. **(B)** miRNA in fibrosis. Several miRs have been described as regulators of cardiac fibrosis acting as pro-fibrotic or anti-fibrotic factors on ECM remodeling (black) and on TGFβ /Smad signaling (blue).**^1^**Wei et al. (2013). **^2^**Wang et al. (2015). **^3^**Thum et al. (2008); Roy et al. (2009), Liang et al. (2012); Dong et al. (2014), He et al. (2016). **^4^**Bernardo et al. (2012); Huang et al. (2014). **^5^**Du et al. (2016). **^6^**Zou et al. (2016). **^7^**van Rooij et al. (2008), Abonnenc et al. (2013), Zhang et al. (2014). **^8^**Duisters et al. (2009), Castoldi et al. (2012), Chen et al. (2014), Muraoka et al. (2014), Wang et al. (2016). **^9^**Li et al. (2016). **^10^**Tijsen et al. (2014). **^11^**Hong et al. (2016). **^12^**Pan et al. (2012). **^13^**Beaumont et al. (2014).

Sphingosine 1-phosphate is the intermediate breakdown product of the catabolism of complex SLs (**Figure 1**), a class of lipids characterized by a C8 carboamide alcohol backbone that was discovered in the brain in 1870 (Thudichum, 1884). S1P is present in the plasma, where it binds to ApoM on HDL particles, and serum albumin (Christoffersen et al., 2011). This bioactive lipid is in all types of mammalian cells (Hannun and Obeid, 2008) and, systemic and local gradients of S1P are essential for immune cell homing (Olivera et al., 2013; Nishi et al., 2014). S1P is formed from Sph by two differently localized and regulated enzyme isoforms, SphK 1 and SphK2 (Maceyka et al., 2012). Although SphK1 and SphK2 catalyze the same reaction, SphK1 inhibition/gene ablation decreases blood S1P, while SphK2 inhibition/gene ablation increases blood S1P. At the cellular level, studies have shown the involvement of SphK1 in cell survival and cell growth, whereas SphK2 is rather associated with growth arrest and apoptosis (Liu et al., 2003).

Sphingosine 1-phosphate acts inside cells as a signaling molecule that regulates specific targets (Maceyka et al., 2012), such as PHB2, a highly conserved protein that regulates mitochondrial assembly and function, TRAF-2, which is upregulated in fibroblasts, and NF-κB, which is crucially involved in inflammatory gene regulation (Xia et al., 2002; Alvarez et al., 2010).

However, in response to only partially known stimuli, S1P can be transported outside the cells by a specific S1P transporter, named Spns2, and upon binding to one or more of the five subtypes of G-protein-coupled receptors (GPCRs), named S1PR1-5, it triggers many downstream signaling pathways (Zu Heringdorf et al., 2013; Kihara et al., 2014; Nishi et al., 2014) (**Table 1**). Hla and Maciag (1990), by a differential display method, discovered the orphan GPCR Edg-1, and successively identified as a S1PR1 receptor based on the sequence homology with LPA1/Vzg-1/Edg-2. Later, other receptors, including Edg-5 and Edg-3, followed by Edg-6 and Edg-8 (now termed S1PR2, S1PR3, S1PR4 and S1PR5), were described (An et al., 1997, 2000; Goetzl et al., 1999).

**Table 1:** Sphingosine 1-phosphate receptors and their intracellular signaling pathways and functions.

S1PR	Knock out phenotype	Intracellular mediators	Biological effects	Cardiac function	Agonists	Antagonists	Indications	Clinical trial
**S1PR1 (Edg1)** Most tissues **Kd (nM)** (8–20) **Coupled to:** Gαi	Normal until E11.5 then lethal, Liu et al., 2000 abnormal yolk sacs, defective blood and smooth muscle, vessels maturation	(-) AC (+) ERK,Rac, PI3K, Akt	Angiogenesis Lymphocytes migration Cardiomyocytes survival	S1PR1 agonist: Improves cardiac function following myocardial infarction S1PR1 antagonists Stroke and ischaemic pre- and post-conditioning cardioprotection	SEW2871 FTY720-P KRP203 BAF312 ACT-128800 ONO-4641	VPC23019 VPC44116 W416	Multiple Sclerosis Crohn’s disease Polymyosites and Dermatomyosites Lupus erythematosus	FTY720-P FDA approved ACT-12880 Phase II ONO-4641 phase II CS-0777 Phase I CYM-5442 Phase III BAF-312 Phase II KRP-203 Phase III
**S1PR2 (Edg5)** Most tissues **Kd (nM**) 16–27 **Coupled to:** Gα_i_, Gα_q_ et Gα_12/13_	Normal until 3–5 weeks, Kono et al., 2004 Excitability at cortical neurons, severe inner ear defects heart development	(-)AC (+) AC, PLC p38MAPK Rho	Vestibular funtion Vascular tone (contraction) Neuronal excitability Cardiomyocyte survival		Not identified	JTE013		
**S1PR3 (Edg3**) Heart Lung Spleen Kidney Intestine **Kd (nM**) 23–26 **Coupled to:** Gαi/o, Gαq, and Gα12/13	Normal Smaller litter size, Ishii et al., 2001	(-)AC (+) ERK, Rac, eNOS, PLC, Akt	Cardiac rytm regulation Vascular tone (relaxation) Cardiomyocytes survival	S1PR3 antagonists Inhibits the S1P-mediated reduction in coronary flow in perfused rat hearts Partially inhibits FTY720-induced bradycardia in rats *in vivo*	FTY720-P KRP203	VPC23019 CAY1044		
**S1PR4 (Edg6)** Lymphoid tissues Blood cells Lung smooth muscle **Kd (nM**) 12–63 **Coupled to:** Gα_i_ and G_1_α_2/13_	Normal, Gräler et al., 1998; Schulze et al., 2011	(+) AC, ERK, PLC, Rho	Megakaryocyte Differentiation Vascular tone (contraction)		FTY720-P KRP203	Not identified		
**S1PR5 (Edg8)** Brain Skin natural killer cells **Kd (nM**) 2–6 **Coupled to:** Gα_i_ and Gα_12/13_	Normal Aberrant natural killer cells, Im et al., 2001	(-) AC, ERK, (+)JNK	Oligodendrocytes survival Mielinization process		FTY720-P KRP203 BAF312	Not identified	Polymyosites and Dermatomyosites	BAF-312 Phase II

*S1P binds to their specific GPCRs, which activate heterotrimeric G-proteins (defined here by their α subunits) to initiate signaling cascades leading to specific biological effects and functions in cardiac tissue. Agonists and antagonists for each specific S1PR subtypes are reported together with the clinical trials in which the drug have been successfully used. AC, adenylate cyclase; Akt, serine/threonine-specific protein kinase 1; eNOS, endothelial nitric oxide synthase; ERK, extracellular signal-regulated kinase; JNK, c-jun N-terminal kinase; PI3K, Phosphatidylinositol-4,5-bisphosphate 3-kinase; PLC, Phospholipase C; p38MAPK, p38 Mitogen activated protein kinase; Rac, Ras related monomeric GTP hydrolase; Rho GTPase, Ras homolog GTP hydrolase; Kd, dissociation constant; (+) activation; (-) inhibition; Clinical trial: FTY720 (Gilenya, Fingolimod) NCT00662649 and NCT01436643; ACT-128800 (Actelion, Ponesimod) NCT01006265; ONO-4641 (Merck/Ono paharmaceuticals, Ceralifimod) NCT01081782; CS-0777 (Daiichi Sankyo Inc.) NCT00616733; CYM-5442 (Celgene, Ozanimod) NCT02531113; BAF-312 (Novartis, Siponimod) NCT01665144 and NCT00879658; KRP-203 (Novartis) NCT01294774 US National Library of Medicine. https://clinicaltrials.gov.*

Sphingosine 1-phosphate receptors are widely expressed and specifically coupled to distinct G-proteins as reported in **Table 1** (Blaho and Hla, 2014; Kihara et al., 2014; Pyne et al., 2015). Recently, studies on the crystal structure of S1PR1 (Hanson et al., 2012) have indicated that the ligand likely binds the receptor by lateral access (Rosen et al., 2013). Substantial evidence has demonstrated that S1PR-mediated signaling regulates many biological processes, such as cell growth and survival, migration, and adhesion (Maceyka et al., 2012; Kunkel et al., 2013; Proia and Hla, 2015); thus, the impairment of the SphK/S1P/S1PR axis leads to many disorders, including inflammation, fibrosis, and cancer (Schwalm et al., 2013, 2015; Newton et al., 2015; Pyne et al., 2015).

## Cardiac Fibrosis

Cardiac fibrosis is a multistep disorder, which arises due to several circumstances, such as inflammation, ischaemia and senescence. Myocardial integrity is assured throughout life by fibrotic remodeling of cardiac tissue that becomes decisive in the progression of cardiac disease, thus contributing to the high risk of mortality for this disease (Cohn et al., 2000; Kong et al., 2014; Gyöngyösi et al., 2017).

Fibroblasts, the major producers of cardiac ECM (Krenning et al., 2010), provide the initial structural support in the neonatal heart, respond electrically to mechanical stretch and participate in the synchronization of cardiac tissue (Tomasek et al., 2002). In chronic conditions of ischaemia or altered oxygen tension, angiotensin-aldosterone mediated oxidative/redox stress, pro-inflammatory and pro-fibrotic factors activate circulating bone marrow-derived fibrocytes, epithelial cells and resident fibroblasts that adopt an hypersecretory myofibroblast phenotype (Porter and Turner, 2009; van den Borne et al., 2010; Lajiness and Conway, 2014). Myofibroblasts, by acting in an autocrine/paracrine manner, start to overproduce ECM. Accumulation of abundant type I/III fibrillar collagen and a variety of bioactive substances causes stiffening of the heart and decreased cardiac function (Gyöngyösi et al., 2017). Among the factors involved in the initiation and progression of cardiac fibrosis, transforming growth factor β (TGF-β) and the local renin-angiotensin-aldosterone system together with other cytokines, including tumor necrosis factor α (TNF-α), interleukin 6 (IL-6), and endothelin-1 (Davis and Molkentin, 2014; Bomb et al., 2016), trigger many signaling pathways, such as Smad protein and GTP hydrolase (GTPase) activation (Rosenkranz, 2004; Wynn, 2008; Porter and Turner, 2009; Leask, 2010; Creemers and Pinto, 2011; Wynn and Ramalingam, 2012). In cardiac matrix remodeling, other key players include the zinc-dependent matrix metalloproteinases (MMPs) and tissue inhibitors of metalloproteinases (TIMPs) (Tyagi et al., 1993; Nagase et al., 2006; Spinale, 2007; Mishra et al., 2013). Aberrant levels of different MMPs and TIMPs are highly correlated with cardiac fibrosis (Moshal et al., 2005; Ahmed et al., 2006; Spinale et al., 2013). MMP-2 and MMP-9 have distinct spatial and temporal actions in cardiovascular remodeling; MMP-2 is constitutively expressed, whereas MMP-9 is inducible (Mishra et al., 2013). In addition to their role in ECM degradation, MMPs also act on non-matrix molecules, such as growth factors, allowing local induction-activation of signaling pathways which has been demonstrated after MMP-9 ablation (Mishra et al., 2010). Among the TIMP isoforms (TIMP1-4) implicated in cardiac fibrosis (Vanhoutte and Heymans, 2010), the level of TIMP1 increases in diseased hearts (Heymans et al., 2005) and high levels of TIMP4 are found in parallel to inhibition of MMP-9. Modulation of ECM turnover and activation of MMPs and TIMPs are controlled by many factors, including TNF-α, TGF-β and ILs (Parker and Schneider, 1991; Tsuruda et al., 2004; Wynn and Ramalingam, 2012; Gyöngyösi et al., 2017). Among these factors, the hormone peptide relaxin (RLX) is a key regulator of ECM remodeling in reproductive and non-reproductive tissues (Samuel et al., 2007; Du et al., 2010; Nistri et al., 2012). Particularly, RLX inhibits pro-fibrotic cytokines (i.e., TGF-β1) and modulates the accumulation and degradation of ECM that acts on MMPs and TIMPs (Samuel et al., 2007; Bani et al., 2009; Du et al., 2010; Frati et al., 2015). Moreover, recent research has found that epigenetic factors, such as miRs play an important role in tissue remodeling by controlling MMPs and TGF-β/Smad signaling (**Figure 1**) (Care et al., 2007; Roy et al., 2009; Creemers and van Rooij, 2016; Biglino et al., 2017). Notably, miR expression can also be regulated by MMP-9 (Mishra et al., 2010). Therefore, this class of small non-coding RNAs, which inhibits gene expression by binding the 3′ UTRs of target mRNAs, can be crucial for the fibrotic process, acting as either pro-fibrotic or anti-fibrotic factors. Since dysregulation in miR expression has been reported in myocardial fibrosis (Gurha, 2016), miRs may represent a novel therapeutic strategy to counteract the fibrotic changes that occur in cardiac diseases (Wijnen et al., 2013).

## Role for the SphK/S1P Axis And S1PR in Cardiac Fibrosis

Many studies performed in cultured cells as well as in animal models have proposed that S1P possesses cardioprotective effects (Kupperman et al., 2000; Jin et al., 2002; Zhang et al., 2007; Karliner, 2013; Maceyka and Spiegel, 2014). In fact, S1P protects cultured rat neonatal cardiomyocytes from ischaemia-induced cell death (Karliner et al., 2001; Jin et al., 2002). Moreover, mice lacking the enzyme SPL that degrades S1P show reduced sensitivity to ischaemia/reperfusion injury and increased S1P level in both plasma and cardiac tissue (Jin et al., 2011) (**Figure 1**).

Regarding cardiac fibrosis, SphK1 appears to play a relevant role. SphK1 is induced by TGF-β and mediates TIMP-1 upregulation, and siRNA against SphK1 inhibited TGF-β-stimulated collagen production (Yamanaka et al., 2004; Gellings Lowe et al., 2009). Importantly, the neutralization of extracellular S1P with a specific anti-S1P antibody significantly reduced TGF-β-stimulated collagen production, indicating the involvement of an “inside-out” signaling of S1P after SphK1 activation in the pro-fibrotic action (Gellings Lowe et al., 2009). Moreover, apelin, an adipocyte-derived factor, inhibits TGF-β-stimulated activation of cardiac fibroblasts by reducing SphK1 activity (Pchejetski et al., 2011).

Notably, high S1P production/accumulation in cells is deleterious. In fact, transgenic mice that overexpressed SphK1 at high level develop spontaneous cardiomyocyte degeneration and fibrosis (Tao et al., 2007; Takuwa et al., 2010) and are characterized by increased levels of Rho GTPases and phospho-Smad3, suggesting that these pathways are downstream of SphK1/S1P.

Recently, we have reported that SL metabolism can be activated by RLX at concentrations similar to those previously reported to elicit specific responses in cardiac muscle cells (van der Westhuizen et al., 2008). In both neonatal cardiac cells and H9C2 cells, RLX induces the activation of SphK1 and S1P production. The silencing and pharmacological inhibition of SphK1 alters the ratio MMPs/TIMPs, and CTGF expression elicited by RLX indicates that hormone peptides promote an ECM-remodeling phenotype through the activation of endogenous S1P production and SM metabolism (Frati et al., 2015).

The role of SphK2 in heart tissue is less clear. Previous research has shown that maternal-zygotic SphK2 is fundamental for cardiac development in zebrafish (Hisano et al., 2015). Moreover, SphK2 knockout sensitizes mouse myocardium to ischaemia/reoxygenation injury (Vessey et al., 2011), and mitochondria obtained from Sphk2 knockout mice exhibit decreased oxidative phosphorylation and increased susceptibility to permeability transition, suggesting a role as protective agent (Gomez et al., 2011). SphK2 appears to be less involved in tissue fibrosis than SphK1 (Schwalm et al., 2015). For example, protein expression of SphK1, but not SphK2, was significantly elevated in lung tissues from patients with idiopathic pulmonary fibrosis (Huang et al., 2013). Presently, the role of SphK2 in cardiac fibrosis is still unclear. Interestingly, Hait et al. (2009) have found that SphK2 is associated with histone H3, and endogenous S1P that is formed in the nucleus via SphK2 inhibits the action of HDACs (Hait et al., 2009; Ihlefeld et al., 2012). Since HDAC activity is increased in patients with cardiac fibrosis (Liu et al., 2008; Pang and Zhuang, 2010), there is a potential link between SphK2, nuclear S1P and the epigenetic regulation of gene expression that is involved in cardiac fibrosis.

Very recently, a strict correlation between the levels of miRs involved in cardiac fibrosis and SL metabolism has been demonstrated. In fact, SphK, SPT, acid SMase and ceramide synthase 6 (CerS6) can be regulated by several miRs (**Figure 1**). miR-613 and miR-124 inhibit SphK1 (Yu et al., 2017; Zhao et al., 2017). miR-137, miR-181c, miR-9, and miR-29 regulate SPT (Geekiyanage and Chan, 2011). miR-15a modulates acidic SMase (Wang et al., 2015), and miR-101a targets Cer6 (Suzuki et al., 2016) (**Figure 1**). Interestingly, miR release into exosome particles depends on the ceramide-dependent pathway (Kosaka et al., 2010).

Although the importance of SphK/S1P system has been thoroughly reported, very little is known about the S1P/S1PR axis in the context of cardiac fibrosis. Given that specific anti-S1P antibodies significantly reduce TGF-β-stimulated collagen production by interfering with the binding of exogenous S1P to its specific receptors, a few years ago, the role of “inside-out” S1P signaling in the fibrotic process was proposed (Gellings Lowe et al., 2009). Three of the five S1PRs (S1PR-1, -2, -3) are the major subtypes expressed in the heart (Peters and Alewijnse, 2007; Means and Brown, 2009). Major candidates for the exogenous S1P-mediated control of the fibrotic process are S1PR2 and S1PR3 (Takuwa et al., 2008, 2010, 2013) that preferentially mediate the two following parallel signaling pathways crucially involved in the fibrotic process: Ras homolog GTPase/Rho-associated-protein kinase (Rho/ROCK) and Smad proteins. Particularly, S1PR3 promotes the activation of Rho signaling and the transactivation of TGF-β (Theilmeier et al., 2006; Takuwa et al., 2010). Under chronic activation of SphK1/S1P signaling, S1PR3 mediates pathological cardiac remodeling through ROS production (Takuwa et al., 2010). Moreover, S1PR3-mediated Akt activation protects against *in vivo* myocardial ischaemia-reperfusion (Means et al., 2007). Characterization of S1PR3-deficient mice also indicates that HDL and S1P promote cardiac protection through nitric oxide/S1PR3 signaling, and exogenous S1P induces intracellular calcium increase through the S1PR3/PLC axis (Theilmeier et al., 2006; Fujii et al., 2014).

Furthermore, S1PR3 can mediate cardioprotection in Langendorff-perfused mouse hearts against ischaemia/reperfusion injury via Rho/NFkB signaling (Yung et al., 2017). Although, S1PR3 is the most prevalent subtype in cardiac fibroblasts (Takuwa et al., 2013), myofibroblast differentiation and collagen production are mainly mediated by S1PR2 signaling (Schwalm et al., 2013). In fact, the silencing of S1PR2, but not of S1PR1 or S1PR3, can block S1P-mediated α-SMA induction (Gellings Lowe et al., 2009), and S1PR2 knock out mice show reduced fibrosis markers expression (Ikeda et al., 2009).

In the heart, the signaling pathways downstream of S1PR1 inhibit cAMP formation and antagonize adrenergic-mediated contractility activation (Means and Brown, 2009). Through S1PR1, S1P induces hypertrophy of cardiomyocytes *in vitro* (Robert et al., 2001) and decreases vascular permeability (Camerer et al., 2009). In bleomycin-induced injury, S1PR1 functional antagonists increase the pro-fibrotic response (Shea et al., 2010), suggesting an antifibrotic action of S1PR1 in the lung tissue.

There is evidence showing that S1P exhibits cross-talk with pro-fibrotic signaling pathways, such as TGF-β (Xin et al., 2004) and PDGF (Alderton et al., 2001). The involvement of S1PRs in the pro-fibrotic effects that are mediated by the cross-talk between S1P and TGF-β has been demonstrated by inhibition of this effect in primary cardiac fibroblasts by the murine anti-S1P antibody, Sphingomab (Gellings Lowe et al., 2009). Specifically, the role of S1PR3 has been reported in the transactivation of the TGF-β/small GTPases system (Takuwa, 2002; Brown et al., 2006). Evidence has also been provided by a study in which the S1PR1 agonists FTY720 mimicked TGF-β action by promoting the differentiation of fibroblasts to myofibroblasts, but failed to act on S1PR3^-/-^ fibroblasts (Keller et al., 2007). Differently from TGF-β, S1P and FTY720-P do not promote Smad signaling to induce ECM synthesis but rather activate PI3K/Akt and ERK1/2 (Sobel et al., 2013). Moreover, TGF-β2 stimulates the transactivation of S1PR2 in cardiac fibroblasts, and the silencing of TGF-β receptor II or co-Smad4 reduces the upregulation of CTGF expression induced by FTY720-P in mesangial cells (Xin et al., 2006).

Recently, our group has demonstrated that extracellular S1P inhibits the effects of RLX on MMP-9 release and potentiates hormone action on CTGF expression and TIMP-1 expression through a S1PR subtype-mediated signaling (Frati et al., 2015). Although the action of RLX on S1PRs expression is unknown, a transactivation between S1PRs and the RLX-specific receptor RXFP1 is worthy of investigation (Bathgate et al., 2013).

MicroRNAs can regulate S1PRs in several pathological conditions; for example, S1PR1 expression is upregulated by the deregulation of miR-148a, leading to TGF-β-dependent epithelial-mesenchymal transition (Heo et al., 2014). TNF-α significantly increases S1PR2 expression in human endothelial cells by reducing miR-130a level (Fan et al., 2016). No data are currently available on the role of miRs that are involved in cardiac fibrosis and S1PR expression.

## S1PR Modulators in Cardiac Functions

### S1PR1 Agonists

**Fingolimod (FTY729),** synthesized from myriocin, is an immunosuppressive product isolated from *Isaria sinclairii* and has received approval from the Food and Drug Administration and from the European Medicines Agency as a drug for the treatment of MS (Gilenya, Novartis) (Chun and Hartung, 2010; Chun and Brinkmann, 2011; Cohen and Chun, 2011). The phosphorylated form, phospho-FTY720 **(FTY720-P)** that is formed by SphK2 *in vivo* (Brinkmann et al., 2010), is a structural analog of S1P that binds and activates S1PR1-3-4-5, but not S1PR2. Notably, the long-term activation of S1PR1 by FTY720-P determines receptor internalization and degradation, thus acting as a S1PR1 antagonist (Graeler and Goetzl, 2002; Matloubian et al., 2004; Brinkmann et al., 2010; Gonzalez-Cabrera et al., 2012). Due to the binding to various S1PRs, FTY720-P is responsible for several collateral effects on MS patients, such as cardiac effects (bradycardia and atrioventricular block) (Sanna et al., 2004; Camm et al., 2014; Gold et al., 2014). Such effects have been attributed to the activation of S1PR3, and in some cases, to S1PR1. Long-term S1PR1 down-regulation contributes to the disruption of Ca^2+^ homeostasis and attenuation of ischaemic preconditioning (Keul et al., 2016).

The phosphorylated form of Fingolimod takes part in cardioprotection in heart transplantation related ischaemia-reperfusion (*I/R*) injury (Santos-Gallego et al., 2016) and acting as potent anti-inflammatory (Aytan et al., 2016) and anti-oxidant agents may lead to reduce myocardial damage as a consequence of reduced cardiomyocytes death.

A new selective S1PR modulator, **ceralifimod (ONO-4641)**, has been recently designed and tested for its ability to limit the cardiovascular complications of Fingolimod (Krösser et al., 2015). Similarly, **Amiselimod (MT-1303)**, a second-generation S1PR modulator, has potent selectivity for S1PR1 and S1PR5 and has almost fivefold weaker GIRK activation (G-protein-coupled inwardly rectifying potassium channel) than FTY720-P (Sugahara et al., 2017). Other interesting compounds that can act on cardiac functions have been reviewed elsewhere (Vachal et al., 2006; Guerrero et al., 2016; Xiao et al., 2016) (**Table 1**). **SEW2871** is structurally unrelated to S1P, and its phosphorylation is not required for binding to S1PR1. SEW2871 promotes lymphopenia by reducing inflammatory cells, especially CD4^+^ T cells (Lien et al., 2006), attenuates kidney ischaemia/reperfusion injury (Lai et al., 2007), and improves cardiac functions following myocardial infarction (Yeh et al., 2009). However, some evidence has indicated that SEW2871 can exacerbate reperfusion arrhythmias (Tsukada et al., 2007). SEW2871 and **AUY954,** an aminocarboxylate analog of FTY720 (Zhang et al., 2009), directly prevent allograft rejection in rat cardiac transplantation through the regulation of lymphocyte trafficking (Pan et al., 2006). Moreover, AUY954 significantly inhibited expressions of IL-17 and MMP-9 in rat sciatic nerves (Zhang et al., 2009). Repeated AUY954 administration enhanced pulmonary fibrosis by inducing vascular leak (Shea et al., 2010), suggesting caution in the use of this drug. **CYM-5442,** which binds to S1PR1 in a structural hydrophobic pocket different from Fingolimod (Gonzalez-Cabrera et al., 2008), induces lymphopenia and promotes eNOS activation in endothelial cells, thus playing a role in vascular homeostasis (Tölle et al., 2016). **Compound 6d** lacks S1PR3 agonism and induces lymphopenia with reduced collateral effects on the heart (Hamada et al., 2010). **BAF-312** is a next-generation S1PR modulator, which is selective for S1PR1 and S1PR5 (Fryer et al., 2012), and has species-specific effects on the heart. BAF-312 induces rapid and transient bradycardia in humans through GIRK activation (Gergely et al., 2012). Notably, different doses may be used to limit cardiac effects (Legangneux et al., 2013). **KRP-203** reduced chronic rejection and graft vasculopathy in rat skin and heart allografts (Takahashi et al., 2005), and in an experimental autoimmune myocarditis model, it significantly inhibited the infiltration of immune cells into the myocardium, reducing the area of inflammation (Ogawa et al., 2007). Currently, KRP-203 is undergoing a clinical trial for subacute lupus erythaematosus and in patients undergoing stem cell transplantation for hematological malignancies.

**S1PR1 antagonists** were reported to have therapeutic potential, but their use requires attention. **VPC23019,** acting as S1PR1 and S1PR3 antagonist, has been used in ischaemic pre- and post-conditioning cardioprotection that is promoted by endogenous S1P in *ex vivo* rat hearts (Vessey et al., 2009). **W-146,** which was initially reported to increase the basal leakage of the pulmonary endothelium (Sanna et al., 2006), has been used to demonstrate the role of the S1P pathway in TGF-β1-induced expression of α-SMA in human fetal lung fibroblasts (Kawashima et al., 2012).

### S1PR2 Agonists

S1PR2 agonists are mainly used in the treatment of hearing loss (**Table 1**). A study has shown that **CYM-5478** has vascular effects, which is indicated by enhanced ischaemia-reperfusion injury *in vivo* (Satsu et al., 2013).

### S1PR2 Antagonists

**JTE-013** was initially reported to affect coronary artery contraction (Ohmori et al., 2003). At long-term S1PR2 antagonism induces several collateral effects, such as a high incidence of B cell lymphoma (Cattoretti et al., 2009).

### S1PR3 Antagonists

Selectively blocking S1PR3 is very difficult. For example, **VPC25239** antagonizes both S1PR3 and S1PR1, affecting smooth muscle cell functions, whereas VPC01091 leads to neointimal hyperplasia by preferentially blocking S1PR3 (Wamhoff et al., 2008). Other antagonists for S1PR3 are as follows: **CAY10444 (or BML-241)** (Koide et al., 2002) that can inhibit the prosurvival effect of HDLs after hypoxia-reoxygenation and **TY-52156** that suppresses the bradycardia induced by FTY-720 *in vivo* and promotes vascular contraction (Murakami et al., 2010).

The therapeutic use of monoclonal antibodies can be a valid alternative to synthetic compounds. A **monoclonal antibody, 7H9,** functionally blocks S1PR3 activation both *in vitro* and *in vivo* (Herr, 2012), reduces the growth of breast cancer tumors and prevents systemic inflammation, representing an effective approach against the morbidity of sepsis (Harris et al., 2012). To date, no specific antagonists for S1PR4 and S1PR5 are available, although (*S*)-FTY720-vinylphosphonate acts as a pan-antagonist that fully antagonizes S1PR1, S1PR3 and S1PR4 and partially antagonizes S1PR2 and S1PR5 (Valentine et al., 2010).

## Conclusion

Targeting S1P signaling might be an intriguing new strategy for the treatment of cardiac fibrosis. However, the double face of the “sphinx” should be carefully considered, and the potential of the multiple collateral effects of S1PR modulators should be evaluated with caution.

## Author Contributions

EM and LM have partecipated in design the main structure of the minireview and in the revision of literature and in the preparation of the text. AV, FP, and AF have participated in reviewing the literature and writing the manuscript and prepare the figure.

## Conflict of Interest Statement

The authors declare that the research was conducted in the absence of any commercial or financial relationships that could be construed as a potential conflict of interest.

## Abbreviations

3KR: 3-keto reductase
AC: adenylate cyclase
Akt: serine/threonine-specific protein kinase 1
ApoM: apolipoprotein M
C1P: ceramide-1-phosphate
cAMP: cyclic adenosine monophosphate
CDase: ceramidase
Cer: ceramide
CerK: ceramide kinase
CerS: ceramide synthase
CTGF: connective tissue growth factor
DeS: desaturase
ECM: extracellular matrix
Edg-1: endothelial differentiation gene-1
eNOS: endothelial nitric oxide synthase
ERK1/2: extracellular signal-regulated kinase 1/2
GIRK: G protein-coupled inwardly rectifying potassium channels
GPCR: G-protein coupled receptors
GTP: guanosine triphosphate
HDAC: histone deacetylase
HDL: high density lipoproteins
IL: interleukin
LPA1: lysophosphatidic acid receptor 1
miR: microRNA
MMP: matrix metalloproteinase
MS: multiple sclerosis
NF-κB: nuclear factor kappa-light-chain-enhancer of activated B cell
PDGF: platelet-derived growth factor
PHB2: prohibitin 2
PI3K: phosphatidylinositol-4,5-bisphosphate 3-kinase
PLC: phospholipase C
Rho GTPase: Ras homolog GTP hydrolase
RLX: relaxin
ROCK: Rho associated-protein kinase
RXFP1: relaxin/insulin like family peptide receptor 1
S1P: sphingosine 1-phosphate
S1PR: S1P receptor
siRNA: short interfering RNA
SL: sphingolipid
SM: sphingomyelin
SMA: smooth muscle actin
Smad: small mother aganist decapentaplegic
SMase: sphingomyelinase
SMS: sphingomyelin synthase
Sph: sphingosine
SphK: sphingosine kinase
SPL: S1P lyase
Spns2: Spinster 2 (S1P transporter)
SPPase: S1P phosphatases
SPT: serine palmitoyl transferase
TGFBR: TGF receptor
TGFβ: transforming growth factor β
TIMP: tissue inhibitors of metalloproteinase
TNF: tumor necrosis factor
TRAF-2: TNF receptor-associated factor 2
UTR: untranslated region
Vzg-1: ventricular zone gene-1.
